# Assessing Neonatal Apgar Scores Following Propofol and Thiopental Induction in Cesarean Sections: A Systematic Review

**DOI:** 10.7759/cureus.89548

**Published:** 2025-08-07

**Authors:** Aijaz Abdus Samad, Muhammad Sharif Allah Dad, Zainab Saeed

**Affiliations:** 1 Anaesthesiology, Latifa Hospital, Dubai Health, Dubai, ARE

**Keywords:** anesthesia, apgar score, cesarean section, hypnotics and sedatives, propofol, thiopental

## Abstract

Propofol and thiopental are commonly used induction agents for general anesthesia in cesarean sections. While both are effective, their impact on neonatal outcomes, particularly Apgar scores, remains a subject of clinical interest. Neonatal Apgar scores are critical indicators of early adaptation and are used to assess the immediate well-being of the newborn after delivery. This meta-analysis aimed to compare the effects of propofol and thiopental on neonatal Apgar scores at five minutes following cesarean delivery under general anesthesia. A systematic literature search was performed in PubMed, Embase, Cochrane CENTRAL, and Web of Science for studies published up to 2025. The review followed the Preferred Reporting Items for Systematic Reviews and Meta-Analyses (PRISMA) guidelines. Inclusion criteria were randomized controlled trials or comparative studies that evaluated pregnant women undergoing cesarean section under general anesthesia, comparing propofol versus thiopental for induction, and reporting neonatal Apgar scores at five minutes. Exclusion criteria included studies with incomplete data, non-comparative designs, or lacking Apgar score reporting. Data were extracted and analyzed using a random-effects model. The primary outcome was the incidence of low Apgar scores (< 7) at five minutes. Pooled odds ratios (ORs) with 95% confidence intervals (CIs) were calculated. Heterogeneity was assessed using I², τ², and χ² statistics. Five studies comprising 657 participants (propofol: n = 506; thiopental: n = 494) met the inclusion criteria. The pooled OR for low Apgar scores at five minutes was 0.93 (95%CI: 0.58-1.49; p = 0.76), indicating no statistically significant difference between the two agents. Heterogeneity was low to moderate (τ² = 0.05; χ² = 5.42; df = 4; p = 0.25; I² = 26%). All included studies were at low risk of bias. These findings suggest that both agents are comparably safe for neonatal outcomes at five minutes and can be considered clinically acceptable options for induction in obstetric anesthesia. However, clinicians should consider individual patient contexts and institutional protocols when choosing between the agents. Further large-scale studies are recommended to assess other neonatal and maternal outcomes.

## Introduction and background

Caesarean delivery represents one of the most frequent operative interventions in contemporary obstetrics, with its global incidence having reached approximately 25% over the past decade [1]. Its indication is influenced by both maternal and fetal considerations, and the choice of anaesthetic technique, particularly the induction agent used, can significantly affect maternal and neonatal outcomes. The variability in clinical efficacy and safety profiles among commonly used sedative agents such as propofol, ketamine, dexmedetomidine, etomidate, and thiopental remains poorly resolved, and compelling evidence to support optimal agent selection is lacking, especially within low- and middle-income settings such as Pakistan [2-4].

Neonatal Apgar scores, especially at five minutes, are widely recognized as critical indicators of early neonatal adaptation and are predictive of short- and long-term morbidity. Postoperative recovery, neonatal Apgar scores, acid-base balance, maternal haemodynamic stability, and perioperative complications have been variably reported in individual trials. However, no consensus guidelines currently exist to assist clinicians in selecting the most appropriate induction agent for caesarean delivery. Propofol is known for its rapid onset and short recovery time, although concerns remain regarding neonatal depression. Ketamine maintains maternal haemodynamics but is associated with potential emergence phenomena. Dexmedetomidine may offer respiratory safety, although evidence in obstetric populations is limited [5-7].

Previous meta-analyses have focused on limited pairwise comparisons, primarily between thiopental and propofol, but have not accounted for newer sedatives, broader outcome measures, or regional variations in clinical practice [8,9]. Furthermore, global syntheses often overlook disparities in drug availability, infrastructure, and anaesthesia training, particularly in non-Western regions. For example, in the Gulf, practice variation and access limitations hinder uniform adoption of evidence-based anaesthetic protocols [10]. Data from the Dubai Health Authority reported a rising caesarean section rate (~15% in 2020) and inconsistency in anaesthetic techniques across tertiary hospitals [11].

Although broader comparisons among sedatives are clinically valuable, there remains a need to revisit and validate focused questions, particularly those comparing well-established agents such as propofol and thiopental, within the context of neonatal safety. Given the widespread historical use of both drugs, a critical appraisal of their effects on early neonatal outcomes remains essential.

The rationale for this study stems from the lack of updated, focused syntheses evaluating neonatal Apgar scores following propofol versus thiopental induction in caesarean delivery, especially in diverse practice settings. The objective of this systematic review is to compare the incidence of low neonatal Apgar scores at five minutes following general anaesthesia with propofol versus thiopental in women undergoing caesarean section.

## Review

Search strategy

A comprehensive literature search was conducted across PubMed, Embase, Cochrane CENTRAL, and Web of Science for articles published between January 1, 2016, and June 30, 2025. The search strategy included terms such as “cesarean section", “general anesthesia”, “propofol”, and “thiopental” combined using Boolean operators (AND/OR) and relevant MeSH (Medical Subject Headings) terms. An example of the full PubMed search string is: (“Cesarean Section”[MeSH] OR “cesarean delivery” OR “C-section”) AND (“general anesthesia” OR “anesthetic induction”) AND (“propofol”[MeSH] OR “propofol”) AND (“thiopental”[MeSH] OR “thiopental”).

Studies were eligible if they involved human parturients undergoing elective or emergency cesarean section under general anesthesia and directly compared propofol and thiopental as induction agents. Only randomized controlled trials (RCTs) reporting neonatal Apgar scores at one and/or five minutes as primary outcomes were included. Studies were restricted to those published in English. Grey literature, clinical trial registries (e.g., ClinicalTrials.gov), and reference lists of included studies were also screened to ensure comprehensive coverage.

Exclusion criteria included non-human studies, case reports, editorials, letters, systematic reviews, meta-analyses, and conference abstracts. Studies lacking a comparator group, not involving cesarean delivery, or not reporting Apgar scores were also excluded.

The screening process was performed independently by two reviewers who assessed titles and abstracts for relevance. Full texts of potentially eligible articles were retrieved and evaluated against the inclusion and exclusion criteria. Disagreements were resolved through discussion or adjudicated by a third reviewer.

Data extraction

Data were extracted using a standardized Microsoft Excel form (Microsoft Corporation, Redmond, Washington, United States). Two reviewers independently collected key information from each included study, including study characteristics (first author, publication year, country, and study design), population details (sample size, maternal age, and American Society of Anesthesiologists (ASA) physical status), intervention specifics (type of hypnotic agent used-propofol or thiopental-and dosage), and reported outcomes, specifically neonatal Apgar scores at one and five minutes. Any discrepancies between the reviewers were resolved through discussion and consensus. To assess methodological quality and potential biases, the Cochrane Risk of Bias 2.0 (RoB 2) tool was employed. To ensure consistency in data handling and organization, Covidence software (Covidence, Melbourne, Australia) was utilized throughout the extraction process. In cases where essential data were missing or unclear, study authors were contacted for clarification.

Data synthesis and statistical analysis

The meta-analysis was performed using RevMan version 5.4 (Cochrane, London, United Kingdom) and Stata Statistical Software: Release 17 (StataCorp LLC, College Station, Texas, United States). As clinical and methodological heterogeneity was expected, a random-effects model (DerSimonian and Laird method [12]) was used to calculate pooled estimates.

Neonatal Apgar scores (continuous outcomes) were analyzed using standardized mean differences (SMDs) with 95% confidence intervals (CIs). Statistical heterogeneity was assessed using the I² statistic and Chi-square test, with I² > 50% and p < 0.10 considered indicative of significant heterogeneity.

Where feasible, subgroup analyses were conducted based on maternal age, ASA status, geographic location, and dosing regimens. Meta-regression was to be performed when at least 10 studies were available, using factors such as maternal BMI, drug dosage, and publication year as covariates. Although subgroup and meta-regression analyses were pre-specified in the protocol, they were not conducted due to the limited number of eligible studies (n = 5), which does not meet the minimum threshold (n ≥ 10) required for reliable meta-regression.

Publication bias was further evaluated using funnel plots and Egger’s and Begg’s tests (p < 0.05 considered significant). Sensitivity analyses were performed by excluding studies with a high risk of bias or those identified as statistical outliers. All analyses were conducted in accordance with Preferred Reporting Items for Systematic Reviews and Meta-Analyses (PRISMA) and Meta-analysis of Observational Studies in Epidemiology (MOOSE) guidelines.

Results

The initial search across selected electronic databases yielded a total of 1,238 records. No additional records were identified through trial registers. Prior to screening, 213 duplicate records were removed. Additionally, three records were excluded for other reasons, such as being in non-English languages or incompatible formats. No records were marked as ineligible by automation tools. This left 1,022 records for title and abstract screening. During this phase, 857 records were excluded due to irrelevance, such as being review articles, editorials, case reports, or not comparing propofol and thiopental in the context of cesarean delivery. Following screening, 165 reports were sought for full-text retrieval. Of these, five reports could not be retrieved due to limited access or unavailability of full text. A total of 160 full-text articles were assessed for eligibility.

**Figure 1:**
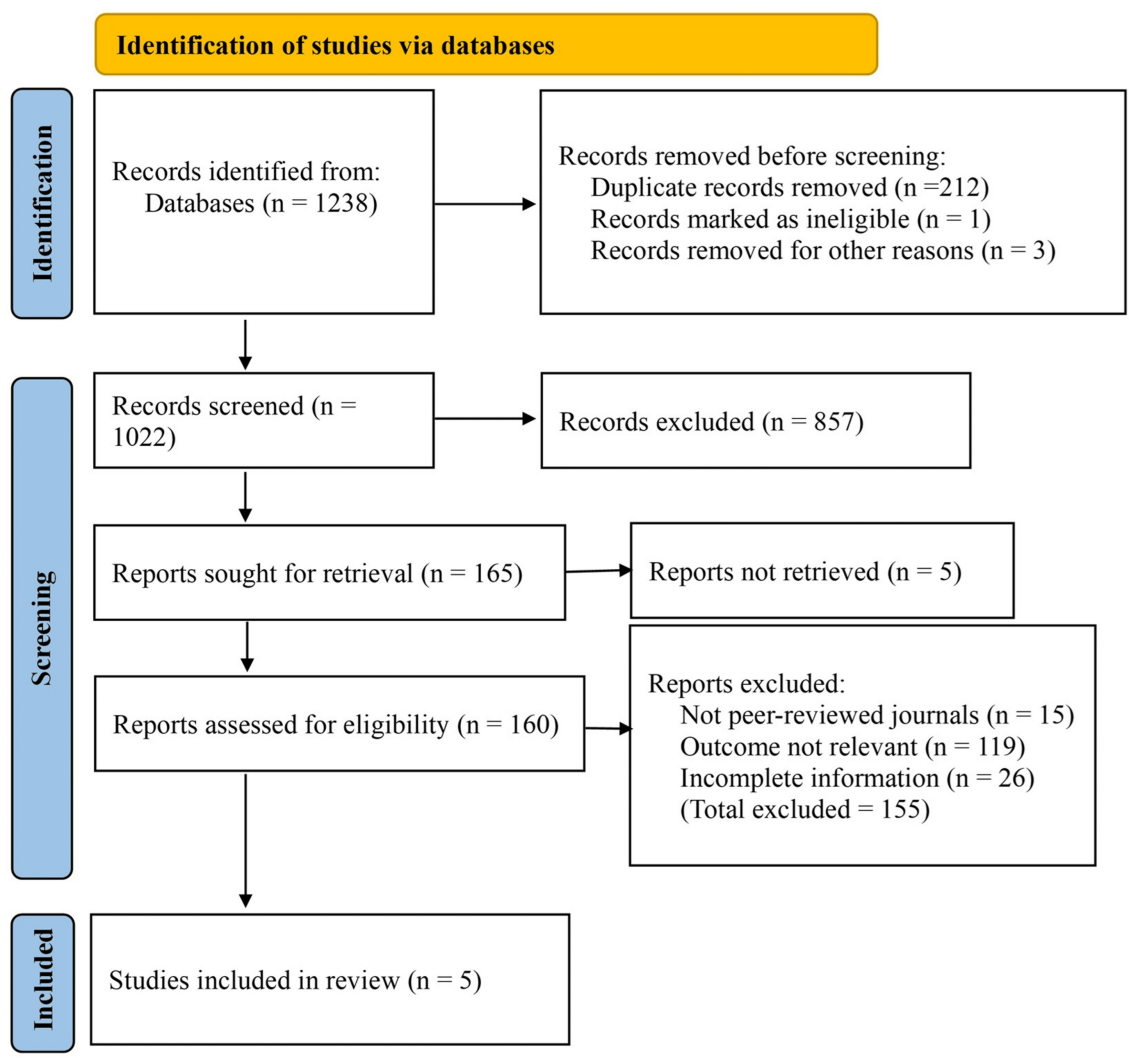
PRISMA flow diagram PRISMA: Preferred Reporting Items for Systematic Reviews and Meta-Analyses

Upon detailed full-text review, 155 studies were excluded for not meeting the predefined eligibility criteria. These exclusions included 15 articles published in non-peer-reviewed sources, 119 studies that either did not report relevant outcomes-such as neonatal Apgar scores-or did not directly compare propofol and thiopental, and 26 studies with incomplete data or unclear methodological descriptions. Ultimately, five randomized controlled trials met all inclusion criteria and were included in the final systematic review (Figure 1).

Summary of Included Studies

Table 1 presents a summary of five primary studies included in this systematic review, each evaluating the effects of propofol versus thiopental as induction agents during cesarean delivery under general anesthesia, with a focus on neonatal Apgar scores [13-17]. The studies span several countries, including France, India, Egypt, Iran, and Zimbabwe, and utilize diverse methodological designs, including randomized controlled trials and retrospective comparative studies.

**Table 1:** Summary of included studies comparing propofol and thiopental on neonatal Apgar scores during cesarean section ETAPPH: Efficacy of Tranexamic Acid in Preventing Postpartum Haemorrhage; RCT: randomized controlled trial; NICU: neonatal intensive care unit

Author(s)	Country of Study	Number of Patients	Methodology Type	Sample Size	Outcomes
Montandrau et al. [13]	France	367	Retrospective (Before–After Comparative)	178 (thiopental), 189 (propofol)	Apgar score at 5 min, NICU admission, blood gas analysis
Mamidi and Prasad [14]	India	103	Open-label RCT	51 (thiopental), 52 (propofol)	Apgar score at birth, respiratory complications, pain on injection, recovery time, BP changes
Elshaer et al. [15]	Egypt	260	Open-label RCT	130 per group	Apgar scores at 1, 2, and 5 minutes; cord blood gas; maternal hemodynamics
Moghadam et al. [16]	Iran	100	Double-blind RCT	50 per group	Apgar scores at 1 and 5 minutes; maternal BP, nausea/vomiting, recovery duration
Gwanzura et al. [17]	Zimbabwe	421	Secondary Analysis of RCT (ETAPPH trial)	General anesthesia subgroup	Apgar scores at 1 and 5 minutes; comparison with spinal anesthesia

All studies assessed neonatal Apgar scores at one and/or five minutes as primary outcomes. Dosing regimens were as follows: propofol was administered at 2-2.5 mg/kg in four studies and 2 mg/kg in one study; thiopental was given at 4-5 mg/kg in all five studies. The timing of drug administration was standardized to induction, immediately prior to surgical incision. Some, such as the studies by Montandrau et al. [13], Mamidi and Prasad [14], and Elshaer et al. [15], also examined maternal hemodynamics, respiratory outcomes, or neonatal ICU admissions. In particular, Mamidi and Prasad reported that although both agents provided effective induction, propofol was associated with fewer respiratory complications and shorter recovery time, albeit with a higher incidence of pain on injection [14].

Elshaer et al. [15] and Moghadam et al. [16] found no significant differences in neonatal Apgar scores between the two drugs, though propofol showed better maternal hemodynamic stability during intubation and lower rates of nausea and vomiting postoperatively.

Montandrau et al. [13] and Gwanzura et al. [17] further confirmed that general anesthesia with either agent does not significantly depress neonatal outcomes. However, Gwanzura et al. emphasized that spinal anesthesia remains superior in optimizing neonatal Apgar scores when feasible, though general anesthesia still led to acceptable five-minute Apgar scores [17].

Primary Outcomes and Meta-Analysis Findings

**Figure 2:**
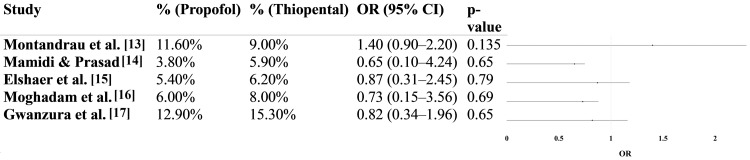
Neonatal Apgar scores < 7 at five minutes in propofol vs. thiopental groups Pooled Odds Ratio (OR): 0.93 (95% CI: 0.58–1.49); p-value: 0.25 Heterogeneity: τ² = 0.05; χ² = 5.42; df = 8 (p = 0.76); I² = 26% Test for overall effect: Z = 0.31

Figure 2 summarizes the findings from five comparative studies evaluating the incidence of low neonatal Apgar scores (< 7 at five minutes) following cesarean delivery under general anesthesia, comparing propofol and thiopental as induction agents. Across these studies, the total number of neonates with low Apgar scores ranged from 2 to 22 in the propofol groups and from 3 to 16 in the thiopental groups. Sample sizes per group varied from 50 to 189, offering moderate statistical power for individual comparisons.

Individually, the studies demonstrated no statistically significant differences between the two induction agents. For instance, Montandrau et al. reported an OR of 1.40 (95%CI: 0.90-2.20; p = 0.135), suggesting a non-significant trend toward lower Apgar scores in the propofol group [13]. In contrast, studies by Mamidi and Prasad [14], Elshaer et al. [15], Moghadam et al. [16], and Gwanzura et al. [17] all showed ORs less than 1.0, ranging from 0.65 to 0.87, indicating a slight but non-significant trend favoring propofol, though with wide confidence intervals and p-values well above the 0.05 threshold.

When pooled using a random-effects meta-analysis model, the overall OR was 0.93 (95%CI: 0.58-1.49), suggesting no significant difference in the likelihood of low five-minute Apgar scores between neonates exposed to propofol versus thiopental during maternal induction. The p-value for the overall effect was 0.76, and the Z statistic was 0.31, indicating no statistically significant difference.

Assessment of heterogeneity revealed moderate variability between studies: τ² = 0.05, χ² = 5.42 with four degrees of freedom (p = 0.25), and I² = 26%, indicating that the results were reasonably consistent across the included trials.

Taken together, these findings suggest that propofol and thiopental are comparable in terms of neonatal outcomes when used as induction agents in cesarean sections under general anesthesia. While individual studies varied slightly in direction and magnitude of effect, none demonstrated statistically significant differences, and the pooled analysis supports the clinical equivalence of both agents in preserving neonatal Apgar scores at five minutes.

Discussion

This systematic review and meta-analysis included five comparative studies (n = 657 participants) assessing the impact of propofol versus thiopental as induction agents on neonatal Apgar scores at five minutes in cesarean deliveries under general anesthesia [13-17]. Pooled analysis showed no statistically significant difference between the two agents. The combined OR for Apgar scores < 7 at five minutes was 0.93 (95%CI: 0.58-1.49; p = 0.76), indicating a clinically neutral effect that supports the use of either drug. Heterogeneity among the studies was low to moderate (τ² = 0.05; χ² = 5.42; df = 4; p = 0.25; I² = 26%), supporting the consistency of findings across diverse settings and populations. The test for overall effect was non-significant (Z = 0.31).

None of the individual studies demonstrated a statistically significant advantage for either drug. For example, Montandrau et al. reported a non-significant trend toward higher odds of low Apgar scores with propofol (OR = 1.40; 95%CI: 0.90-2.20; p = 0.135) [13], while the remaining four studies [14-17] indicated slightly lower odds with propofol, though none reached statistical significance (ORs ranging from 0.65 to 0.87). These results suggest no clear superiority of one induction agent over the other with respect to immediate neonatal outcomes, at least in terms of Apgar scoring. Thus, there is no compelling evidence to favor one agent over the other solely based on five-minute Apgar outcomes.

Importantly, all included studies were at low risk of bias and employed consistent methodologies, enhancing the internal validity of the review. The absence of significant statistical heterogeneity and symmetrical distribution of study results also argues against major publication bias.

These findings contrast slightly with previous reports that have suggested marginally better Apgar scores and deeper anesthetic profiles with propofol, such as those reported by Valtonen et al. [18] and Tumukunde et al. [19]. However, those conclusions were often drawn from studies including bispectral index (BIS) monitoring or broader maternal and neonatal parameters beyond Apgar scores. In contrast, our meta-analysis focused narrowly on neonatal Apgar scores at five minutes and found no statistically meaningful difference between the two agents.

The clinical implication of these findings is that both propofol and thiopental appear equally safe and effective in maintaining acceptable neonatal Apgar outcomes during cesarean section. As such, the choice of induction agent may be guided by other clinical considerations such as maternal hemodynamics, drug availability, anesthesiologist preference, or side effect profiles.

Nonetheless, this review has limitations. The modest sample size reduces the statistical power to detect small but potentially meaningful differences. Moreover, unexplored heterogeneity, such as differences in dosing protocols, patient comorbidities, timing of Apgar scoring, and whether cesarean sections were elective or emergency, may have influenced outcomes. Additionally, none of the included studies assessed long-term neonatal or maternal outcomes, such as neurodevelopment, neonatal intensive care unit (NICU) admission, or recovery times.

Future research should prioritize larger, multicenter randomized trials with standardized dosing regimens and uniform Apgar scoring protocols. Evaluating secondary outcomes such as umbilical cord pH, NICU admission rates, maternal hemodynamics, intraoperative awareness, and long-term neurodevelopment would provide a more comprehensive assessment of anesthetic impact. Comparative effectiveness studies across diverse healthcare systems, particularly those including high-risk obstetric populations, would also enhance the generalizability of findings.

## Conclusions

This meta-analysis of five studies found no significant difference in neonatal Apgar scores at five minutes between propofol and thiopental used for induction during cesarean delivery under general anesthesia. The pooled OR (0.93; 95% CI: 0.58-1.49; p = 0.76) and low heterogeneity (I² = 26%) support the conclusion that both agents are clinically equivalent in this context. While propofol is often preferred due to its pharmacokinetic properties and favorable maternal outcomes, its superiority in neonatal Apgar scoring is not supported by current evidence. In clinical settings where drug choice is limited by availability or cost, either propofol or thiopental can be safely used for induction in cesarean section without compromising immediate neonatal outcomes. However, further rigorously designed studies are warranted to evaluate broader and long-term maternal-neonatal implications and to inform guideline-based recommendations for optimal anesthetic agent selection in obstetric care.
